# Three Years of Continuous Vital Signs Monitoring on the General Surgical Ward: Is It Sustainable? A Qualitative Study

**DOI:** 10.3390/jcm13020439

**Published:** 2024-01-13

**Authors:** Harm H. J. van Noort, Femke L. Becking-Verhaar, Wilmieke Bahlman-van Ooijen, Maarten Pel, Harry van Goor, Getty Huisman-de Waal

**Affiliations:** Department of Surgery, Radboud University Medical Centre, 6500 HB Nijmegen, The Netherlands; femke.becking-verhaar@radboudumc.nl (F.L.B.-V.); wilmieke.vanooijen@radboudumc.nl (W.B.-v.O.); maarten.pel@radboudumc.nl (M.P.); getty.huisman-dewaal@radboudumc.nl (G.H.-d.W.)

**Keywords:** vital signs, clinical deterioration, monitoring, wearable electronic devices, continuous vital sign monitoring

## Abstract

Continuous monitoring of vital signs using a wireless wearable device was implemented in 2018 at a surgical care unit of an academic hospital. This study aimed at gaining insight into nurses’ and patients’ perspectives regarding the use and innovation of a continuous vital signs monitoring system, three years after its introduction. This qualitative study was performed in a surgical, non-intensive care unit of an academic hospital in 2021. Key-user nurses (nurses with additional training and expertise with the device) and patients were selected for semi-structured interviews, and nurses from the ward were selected for a focus group interview using a topic list. Transcripts of the audio tapes were deductively analysed using four dimensions for adoptions of information and communication technologies (ICT) devices in healthcare. The device provided feelings of safety for nurses and patients. Nurses and patients had a few issues with the device, including the size and the battery life. Nurses gained knowledge and skills in using the system for measurement and interpretations. They perceived the system as a tool to improve the recognition of clinical decline. The use of the system could be further developed regarding the technical device’s characteristics, nurses’ interpretation of the data and the of type of alarms, the information needs of patients, and clarification of the definition and standardization of continuous monitoring. Three years after the introduction, wireless continuous vital signs monitoring is the new standard of care according to the end-users at the general surgical ward.

## 1. Introduction

Measuring vital signs is crucial to evaluate the clinical condition of surgical patients. Subtle changes in vital signs such as respiratory rate, blood pressure, or heart rate can be the first signals of clinical deterioration. Nurses have crucial roles in the recognition of patients’ clinical deterioration [1,2]. Nurses estimate modified Early Warning Scores (MEWSs) [3] indicating the clinical risk for clinical deterioration, admission to intensive care units (ICU), or severe events such as cardiac arrest or even death [4,5,6,7,8,9,10]. MEWSs are based on values of the vital signs: respiratory rate, blood oxygen saturation, blood pressure, heart rate, temperature, and level of consciousness. These parameters give the actual state of the physiological wellbeing of patients and indicate the need for medical treatment. Nurses also recognize subtle signs of deterioration by observing the patients and using their clinical judgement [2,10,11]. Nurses develop a sense of worry regarding the situation of their patients in cases of clinical deterioration [12]. While the nurses’ sense of worry is sensitive to adverse events, deviations in vital signs are crucial factors that should be assessed and interpreted accordingly.

Compliance with MEWS protocols can be low, including subsequent follow ups according to the vital sign safety protocol [13,14,15]. Failures to timely escalate treatment still happen because of the insufficient measurement of vital signs [16,17]. Remote devices are introduced for the continuous monitoring of vital signs [18,19]. With the Visi Mobile as a wireless monitoring system, nurses mentioned an earlier identification of clinical decline, enhanced responses to clinical decline, and increased feelings of safety because of the higher frequency of vital signs measurements [20,21,22]. In our hospital, the introduction of the Visi Mobile in 2018 moved practice from the intermittent or periodic measurement of vital signs to continuous wireless monitoring. The Visi Mobile facilitates bedside and from-a-distance monitoring of vital signs by nurses. Since then, a decline in unplanned ICU admissions has been observed [23].

The CeHRes roadmap, a framework to achieve optimal uptake of eHealth technologies, suggests formatively evaluating the actual uptake or usage of technology to improve the technology and its use and to ensure sustainable use of the technology [24]. A post-implementation survey demonstrated that nurses were positive towards the use of the continuous vital signs monitoring system in daily practice [25]. However, understanding the nurses’ and patients’ perspectives for further development of the use of this system is lacking. Therefore, to proceed on the findings of Becking-Verhaar et al. [25], this study undertook a qualitative interview approach to provide in-depth insight into nurses’ and patients’ perspectives regarding the use of a continuous vital signs monitoring system, and how it can be further innovated to ensure the sustainable and effective use of this technology.

## 2. Materials and Methods

### 2.1. Design

This study was framed within the evaluation phase of the CeHRes Roadmap [24]. This study followed a qualitative approach by conducting individual interviews and a focus group interview with relevant stakeholders. This study was conducted between February and April 2021, which was three years after the introduction of the Visi Mobile. The Standards for Reporting Qualitative Research [26] were used to ensure transparent reporting. Furthermore, this study was performed according to the ethical guidelines of the declaration of Helsinki [27]. All participants provided informed consent a priori and data were treated anonymously. Under Dutch law there is no need for a formal ethical review for this type of study.

### 2.2. Setting

This study took place at a gastrointestinal surgical oncology unit of a Dutch academic hospital. In the 18-bed surgical ward, patients mainly recover from major abdominal surgical procedures, such as oesophageal resection, liver resection, pancreatic resection, colorectal surgery, and cytoreductive surgery with hyperthermic intraperitoneal chemotherapy.

#### System for Continuous Monitoring: Visi Mobile

The Visi Mobile (Sotera Wireless) was implemented on the surgical ward in May 2018 after a pilot phase (see Supplementary S1) [20]. The Visi Mobile is a wireless, wearable device with sensors at the thumb and chest for continuous measurement of oxygen saturation, heart frequency, body temperature, respiratory rate, heart rhythm, and blood pressure. These parameters are displayed on screens of the battery worn on the patients’ wrist and on monitors at the nurse stations and lunchrooms [23]. The monitors provide nurses with continuous real-time vital sign data trends of the preceding 96 h and display single channel alarms when a single vital sign falls outside the pre-set safety limits. Vital signs data are automatically sent to electronic health records for automated MEWS calculation with on-demand monitoring by nurses. Nurses use the patients’ data if they deem it to be necessary. Vital signs and the corresponding MEWSs are determined at least three times a day using the Visi Mobile device at the bedside. Nurses intervene depending on the MEWS or alarms given by the devices on the patient’s wrist or on the dashboards. Alarms noted from a distance require evaluation by the nurse. Where alarms are accurate measurements of abnormal values, nurses validate the MEWSs at the bedside. Interventions are based on the hospital’s MEWS protocol and the clinical judgement of the nurse. Key-user nurses were established for the Visi Mobile, having experience and providing supervising roles for other nurses at the unit regarding remote continuous monitoring for coaching and problem solving. They were involved during the initial implementation and were more experienced in the use of continuous monitoring.

### 2.3. Participant Selection

The main stakeholders were end-users of vital signs monitoring using the Visi Mobile at the gastrointestinal surgical oncology unit. The actual users of the system were nurses, key-user nurses, and patients admitted to the surgical unit.

Nurses were selected because they were continuously responsible for the safety of patients. From the nursing staff (n = 35), nurses were recruited for a focus group interview by using email and announcements during regular team meetings. A focus group was the preferred method due to its ability to facilitate optimal interaction between the nurses. Nurses were selected if they had worked for at least six months at the ward, to ensure sufficient experience with the remote continuous monitoring, and were not a key user.

Key-user nurses (n = 4) were recruited for individual interviews to collect in-depth insight into their perspectives on the uptake of the technology. Respondents were contacted face-to-face or by email for participation.

Also, patients were individually interviewed because they wore the device and experienced how nurses used it in daily practice. They were consecutively approached for participation if they were able to provide informed consent, were monitored by the Visi Mobile, understood and spoke the Dutch language, and were in a condition to talk about this topic based on the nurses’ judgement. If they were open to an interview, an appointment was made to hold the interview.

### 2.4. Data Collection

All interviews were held face-to-face in calm and private rooms during the hospital stay of the patient in the afternoon at the patient’s preferred time. Patients wore the particular device during data collection. The interviews with nurses lasted between 45 and 55 min and were conducted by one researcher (MP). The focus group interview took 75 min and was conducted by two researchers, of whom one was the interviewer (HN), and the other the observer (MP). The interviews with patients lasted for approximately 20 min and were undertaken by one of the researchers (MP, HN). MP was a Bachelor of Nursing student during this study and received supervision from HN and FBV. HN is a clinical academic nurse with experience in education, surgical nursing, and qualitative research. FBV is also a clinical academic nurse with experience in surgical nursing and pedagogy. All researchers had experience in individual interviewing, HN was also experienced in focus groups. The researchers discussed, before the first interviews and after each interview, their skills, the topic list, and the responses, to ensure quality of the data collection. All interviews were audio recorded and transcribed verbatim. For each group of respondents, a tailored topic guide was developed by MP, FB-V and HN, focusing on the aim of this study. The topic lists are shown in Supplementary S2.

#### 2.4.1. Individual Interviews with Key-User Nurses

The themes during the interviews were: (a) experiences with continuous monitoring, (b) experiences regarding barriers, (c) possible improvements, and (d) preconditions (see Supplementary S3). The experiences with continuous monitoring were discussed by illustrating the facilitators and barriers to working with the system found in previous studies [20,25]. This enabled the comparison of similarities and differences between the previous and current perspectives, as was suggested by the CeHRes Roadmap [24]. Hence, the device was used for several years in which periodic improvements could have eliminated some barriers. The topic list continued to explore perspectives on possible improvements that could innovate the nurses’ use of the continuous monitoring of vital signs. Therefore, the respondents were probed to suggest directions for solutions and subsequent requirements, and how these solutions could be integrated into daily nursing routines. In this phase, it was emphasized that a new role for a central nurse could focus on all kinds of tasks and activities that are related to the continuous monitoring of vital signs. Each interview ended by asking the respondent to prioritize a main current problem and direction for the solution.

#### 2.4.2. Focus Group Interview with Nurses

The focus group started with attention as to how participants could discuss and interact with each other during the interview about the topics (see Supplementary S2). Then, the same topics as used in the individual interviews with nurses were introduced.

#### 2.4.3. Individual Interviews with Patients

The topic list for patients included: (a) experiences with continuous monitoring, and (b) possible improvements (see Supplementary S2).

### 2.5. Data Analysis

A deductive content analysis approach was applied [28,29], guided by the list of factors that are related to the success or failure of information and communication technologies (ICT) adoption made by Gagnon et al. [30]. These factors are structured within four dimensions with (sub)indicators (see Table 1 and more detailed information in Supplementary S2). These dimensions were previously used to determine the feasibility of the Visi Mobile device [20] and were therefore used to explore what can be further innovated within each dimension. Two of three researchers (MP, HN, and FB-V) independently attributed the data of the transcripts to the dimensions and to the (sub)indicators of each dimension. Consensus was reached about the attribution of each citate afterwards. Then, data were summarized per indicator of the dimensions.

## 3. Results

Individual interviews were held with four key-user nurses and five patients. One of the four key-user nurses was male, all other respondents were female. Of the five patients, three were male. Six female nurses participated in the focus group interview. For the dimension ‘Factors related to the ICT’, citations were only attributed to the indicators ‘design and technical concerns and characteristics of the innovation’. For the dimension ‘Individual factors of healthcare professionals’, citations were attributed to the indicator’s ‘knowledge’ and ‘attitude’. For the dimension of ‘Human environment’ and ‘Organizational aspects’, citations were attributed to both indicators. No data were attributed to the remaining indicators of the dimensions. The results of the analysis are demonstrated per attributed indicator. Citations are displayed in Table 2.

### 3.1. Factors Related to ICT

#### 3.1.1. Design and Technical Concerns

The nurses recognized several barriers that still affected the use of the continuous monitoring system. In particular, technical disturbing aspects were mentioned that required improvements. Disturbing aspects were clustered into four main aspects: (1) the connectivity between the device and the WIFI, (2) usability of the battery and cables, (3) reliability of the estimations, and (4) the amount of data that the system provides. Regarding the first aspect, the WIFI sometimes did not connect, which made it impossible to load the data from the electronic patient file. Secondly, patients mentioned that the battery was too big and heavy, and turned around on their wrist, which hampered the patient in daily activities. The battery had to be charged twice a day, which sometimes led to an empty battery and unavailable vital signs. Thirdly, nurses also mentioned that incorrectly placed cables (i.e., the ECG-leads, the thumb sensor, and the electro lead) led to measurement errors and false alarms. Moreover, some nurses described that patients’ blood pressure measured by the Visi Mobile was not always a reliable estimation of the parameter. The blood pressure seemed to be less accurate the further away from calibration. Therefore, the reliability of estimations of the vital signs had to be improved, according to some nurses. Fourthly, the amount of data that continuously monitoring provides for nurses could be seen as an overload of data. The amount of data enables nurses to make trend analyses of vital signs such as falling blood pressure or a rising heart rate. Nurses indicated that they used trends to consult a physician. Some nurses explained that they learned how to handle the data overload over time by prioritizing and being aware of the clinical condition of the patient. Some of them also mentioned that the trend analysis was still far from optimal and may be improved using artificial intelligence. This could be helpful to deal with the overload of patient data and the use of trends for the recognition of clinical decline.

#### 3.1.2. Characteristics of the Innovation

Nurses perceived continuous monitoring as an extra set of eyes of the nurse, enabling them to provide better care. Nurses did not have to be at the bedside the whole time in case of a sense of worry. The extra set of eyes provided feelings of trust. However, if a nurse was busy with a particular patient, she was not warned in case of abnormal vital signs of another patient. Some nurses mentioned this as a feeling of uncertainty. Moreover, some nurses stated they did not dare to let patients sleep without monitoring their vital signs continuously. Before the introduction of continuous monitoring, vital signs were measured three times a day (or more often, depending on the MEWS). Nurses described that deterioration of patients during night shifts was only noticed during the morning rounds. With the use of continuous monitoring, deterioration during nightshifts is recognized earlier, and interventions can be applied, which improves patient outcomes, according to nurses.

Patients also described that they felt safe due to the idea of their vital signs being monitored continuously. Some of them experienced a nurse coming to the bedside in case of abnormalities. They now knew that their vital signs were being monitored and that subsequent action would be taken.

### 3.2. Individual Factors of Healthcare Professionals

#### 3.2.1. Knowledge

The nurses described that they gained knowledge about working with continuous monitoring. One of the nurses reported that during the introduction of this device (in 2018, red.) nurses immediately responded to an alarm by seeing the patient, also in the case of clear false alarms. One of the nurses mentioned an example of patients sleeping on their right or left side, whereby the Visi Mobile displayed no respiratory rate. Currently (in 2021, red.), nurses first look for the type of alarm and consider all measurements at that moment before acting on an alarm. In the case of alarm for breathing absence, nurses first look at saturation, heart rate, and whether the patient slept on a side (patients’ attitude was measured with VM, red.), before they go to check on the patient. Three years after implementation, the nurses felt they understood all types of and reasons for alarms and that they handled the continuous data availability of vital signs efficiently. To work adequately and efficiently with continuous monitoring, knowledge regarding alarms must be integrated into training for nurses.

#### 3.2.2. Attitude

The nurses felt mainly positive about the use of continuous monitoring, whereby their attitudes had changed over time from a more sceptical attitude to an enthusiastic attitude. Nurses described that CM became an essential part of their care, which facilitated them in anticipating patients’ clinical deterioration. In addition, having patients’ vital signs provided feelings of safety among the nurses. For example, nurses had experienced cases where resuscitation was initiated because of abnormal values of vital signs that came to nurses due to continuous monitoring. Nurses mentioned that the system guarantees more patient safety and better clinical conditions.

The nurses’ attitude was also critical towards the use of trends. Due to the continuously available vital signs, a bored feeling among nurses could arise, which would affect their alertness to changes. When measuring the MEWS three times a day, changes in MEWS were always noted. However, these changes are less explicitly noticed now because vital signs are present all the time. Therefore, some nurses mentioned a critical note towards the possibility of only analysing trends, because small changes may be overlooked. Nurses explained the advantage of observing trends in vital signs, for example, regarding suspected false alarms such as breathing frequency. Thereby, although some nurses really examined the patients’ situation, others only looked at the numbers instead of attending patients for physical examination. One of the nurses described a wish to actively discuss trends during the physicians’ round, because this was currently no part of it. Another factor that affected the nurses’ attitude towards monitoring was over-monitoring. It appeared that continuous monitoring was still used even if vital signs were stable for a long time, for instance, in the case of a patient who was to be discharged. This over-monitoring, measuring vital signs when the clinical added value was not clear, did provoke a tired feeling towards continuous monitoring.

### 3.3. Human Environment

#### 3.3.1. Factors Associated with Patients

Patients had generally positive feelings about the use of continuous monitoring. Patients described that it was good for their health that someone was able to watch over them continuously and that, in case of abnormalities, nurses could respond immediately. Patients were soon used to the device and only noticed it during calibration by the nurse or changing batteries. Patients described increased comfort because blood pressure was measured all the time, while the burden of the blood pressure cuff happened only once in 24 h during calibration. Also, patients mentioned looking frequently at their own vital signs and health status, especially during the first postoperative days. Some patients noticed that they should not look at their screen that often because they interpreted the vital signs according to how they felt. Patients could feel worried when vital signs were going out of normal ranges, and confident after seeing good vital signs. Furthermore, patients indicated the value of interaction with a nurse who knew them, who applied the device, and who interacted with them about the vital signs. Some patients mentioned that the involvement of many different nurses in their care could be difficult because they wanted to be known personally. To further innovate the use of continuous monitoring, this value must be recognized.

Nurses also mentioned that patients might look frequently at their device, indicating that the patients might need some further information or support as to how they can interpret their own vital signs. They expressed the need to investigate how patients use the continuous monitoring system, to be able to supervise them better.

#### 3.3.2. Factors Associated with Peers

The system for continuous monitoring was used by nurses and not by nurse assistants. Nurses stated that nurse-assistants could make an assisting contribution to the measurement of vital signs monitoring. They could change batteries, apply the system, and calibrate the blood pressure. They should be trained in these activities. Also, the nursing team should be properly aware of the expectations concerning continuous monitoring, such as changing responsibilities, policies on how to act on alarms, and whether high-risk medication could be administered while the patient is being monitored. The function of a dedicated nurse could be embedded into daily patient care by having a pager for incoming alarms. Nurses described the dedicated nurse as a partner for dialogue who addresses the practical implications of continuous monitoring, such as changing batteries or pasting stickers. Some nurses felt that such a position would remove their responsibilities and position in handling and responding to clinical decline. The majority described it as an appropriate task for nurse assistants. Therefore, the nurses concluded there was room for improvement through monitoring as a team with nurses and nurse-assistants.

### 3.4. Organizational Aspects

#### 3.4.1. Internal Aspects

Regarding the organization of the use of the continuous monitoring system, nurses mentioned that they were not able to continuously watch the vital signs, as they were not all the time in one of the rooms where the dashboards were available. Therefore, the nurses notified each other in case of abnormal vital signs. In this matter, task-specific nursing in terms of a dedicated nurse was discussed as part of the topic guide. This possible new nursing function could be a supportive role for ward nurses by making them aware of abnormalities in patients’ vital signs. The dedicated nurse first assesses the amount of data and possible false alarms before warning the ward nurses. However, such a role was not perceived as supportive by all nurses. Most nurses indicated that they wanted to have the final responsibility for patients’ vital signs and the total care of patients. They expressed the wish to make a careful assessment themselves with a physical examination of patients and their clinical view before intervening. For newly graduated nurses, the help of a dedicated nurse can be supportive in facilitating a back-up for the nurse when she is unexperienced.

#### 3.4.2. External Aspects

As the general ward is not organized according to intensive care standards, nurses mentioned that there is a difference between continuous monitoring and guarding vital signs. One of the participating nurses described that continuous monitoring provides more of an “own assessment” and “own feeling of concern”. Nurses assess the vital signs of patients and use the system and their clinical judgement to intervene on a deviation. In intensive care facilities, every change is noticed and subsequently handled. Although nurses mentioned that a dedicated nurse for this task could facilitate responses to each alarm, they perceived that this was more guarding vital signs than monitoring vital signs. A clear definition of what continuous monitoring of vital signs at general wards is, and what kind of boundaries are determined, would enhance clarity as to the expectations towards nurses.

## 4. Discussion

This study assessed perspectives on the use of a continuous monitoring system for recognition of clinical decline three years after introduction and how it can be innovated to ensure sustainable use. The perspectives were structured by the four dimensions of Gagnon’s list for adoption of ICT devices in healthcare. Generally, monitoring vital signs with the Visi Mobile enabled nurses to improve patient safety, because they could more frequently review vital signs to anticipate and act sooner on changes in patients’ vital signs. Nurses gained knowledge and skills in using the system for measurement and interpretations over the three years. Patients felt safe while monitored, which seemed to overcome the physical concerns about the device. Although these findings are in line with multiple references [19,20,22,25,31,32], this is the first study that has performed a formal evaluation 3 years after initial introduction which also identifies four areas to ensure sustainable use.

First, the technical aspects of the device can be improved, such as the battery size, battery life, and Wi-Fi. These barriers were previously identified [20,25] and are also important factors for other devices [33]. The technical aspects of devices for vital signs measurement are important to address, with a priority regarding the reliability of measurements. Furthermore, the user-friendly technical aspects are important. Medical companies who produce the devices can now address these daily barriers for future innovation of their devices to improve adoption and the satisfaction of end-users [34,35].

The second opportunity concerns the nurses’ interpretation of the data and alarms. First, false alarms can be reduced by preventing measurement errors. Although it appeared in our findings that nurses gained knowledge over the years, measurement errors leading to false alarms still occurred. This can provoke feelings of irritation and uncertainty towards the system [36]. It is unclear whether these measurement errors are caused by device-related factors or nurse-related factors. Further analysis is required on this topic. Secondly, effective interpretation of all available data and reporting of vital signs or MEWS using trends will enhance the value of the system, according to the nurses in our study. Nurses need to process all the data, which can be seen as overload. The use of algorithms may be a solution to support nurses in the interpretation of data [37,38]. Furthermore, nurses expressed in our study that integration of their clinical judgement into the recognition of clinical decline was pivotal to complementing the data assessed by the Visi Mobile. Nurses’ feelings of worry must be recognized [11] and can be measured [39]. Future studies can address vital signs-based algorithms that are complemented by the nurses’ worries and clinical judgement to improve recognition of clinical decline.

Thirdly, the perspectives of patients should be addressed in performing continuous vital signs monitoring. Patients appreciated interaction with their nurse, in line with previous research [40]. Nurse-patient interaction is the core of fundamental care [40,41], requiring attention in future studies, as remote monitoring might affect this. Furthermore, our results illustrate that the interpretation by patients of their clinical condition requires further attention. Patients expressed the need to be informed about the normal values of the vital signs. Therefore, future research should address the perspectives of patients, to ensure that patients with all levels of health literacy are kept informed and empowered [1]. Furthermore, although our patients felt safe with the device, future research can address the patients’ responses towards the wide range of wireless devices. Finally, patients’ perspectives were integrated as respondents in this study; we suggest, for future developments in continuous vital signs monitoring, partnering with patients in the research group [42].

Fourthly, continuous vital signs monitoring using a wearable, wireless device can be improved by defining and standardizing the use within nursing practice at general wards. In our study, nurses were not always in rooms with the monitors displaying vital signs data, which affected the reality of the term ‘continuous’. Other studies did not use alarms [36], and vitals were interpreted differently [43]. Moreover, our respondents suggested training nurse assistants to administer the device, as they were part of the nursing staff, as well as developing clear guidelines on when to start or stop monitoring. Therefore, clear definition of the concept and practical guidance for these issues will enhance efficacy and prevent over-monitoring.

Our study must be interpreted in the light of several considerations. First, perspectives were explored in one setting using only one device, affecting the generalizability of the findings. Although research on technology in healthcare should address the context, other devices in other settings may lead to different long-term experiences. Secondly, cooperation between nurses and physicians has been previously identified, but not in our findings [11], suggesting that data saturation was not achieved in our study. Future studies must complement our findings regarding the sustainable use of innovations. Finally, we used Gagnon’s list for data analysis, because previous interviews regarding the use of the Visi Mobile were also analysed with this list [20]. Another study in this field used the Behaviour Change Wheel as the theoretical basis of the analysis [44]. Our model enabled the analysis of the sustainable updating of the technology, while their aim was to assess nurses’ behaviours regarding wireless monitoring.

## 5. Conclusions

Three years after the introduction of a continuous vital signs system on a general surgical ward, it enables nurses to ensure patient safety and provides feelings of safety for both nurses and patients. The nurses gained knowledge and skills to handle the system. Nurses and patients provided four opportunities to further improve the use of a continuous vital signs monitoring system. These concern the device itself, to make it more user-friendly, and the way nurses handle the output, including alarms and trends. Also, the patient’s information and psychological needs regarding vital signs and the judgement of their clinical condition is important for nurses to address. Finally, it is important to outline what continuous monitoring is about, when it can be initiated, and what the responsibilities are for whom.

## Figures and Tables

**Table 1:** Factors related to the adoption of ICT application based on the findings of Gagnon et al. (2012).

Dimension	Indicator
Factors related to ICT	Design and technical concerns Characteristics of the innovation System reliability Interoperability Legal issues Validity of the resources Cost issues
Individual factors of healthcare professionals	Knowledge Attitude Socio-demographic characteristics
Human environment	Factors associated with patients Factors associated with peers
Organizational aspects	Internal environment External environment

**Table 2:** Citations of the respondents per indicator.

Indicator	Respondent	Citation
Design and technical concerns	P4	“The device of the Visi Mobile is unfriendly for patients because the battery is too rude, and heavy. It never fits well, turns around my wrist and slides back and forth. It is not comfortable.”
	R2	“In the future, there may be a possibility to apply artificial intelligence to handle and cluster the data overload.”
Characteristics of the innovation	R1	“From a distance, you can estimate how your patient is doing, to some extent. Also, if your patient does not feel well, and you really want to be in the room all the time, which is not possible in a nursing ward, you feel you can better monitor your patient. So yes, it provides me a safe feeling.”
	R3	“Recently, we had transferred a patient from the ICU to our unit, having a MEWS 6 and respiratory unstable. In that case, it would be great if there is someone who keep an extra eye on that patient, because I cannot constantly look to the display, and do not get an alarm on my pager.”
	P3	“Tonight, a nurse entered my room because my oxygen level was too low. I understand the use of continuous monitoring and I think that the need differs for each ward. For me, this was not a minor operation, so I think this is perfect, for me, but also for them [nurses].”
Knowledge	R1	“Nine out of ten times you do not have to respond to a false alarm, but you just wait a few seconds before breathing frequency or saturation will improve. I have the idea we are on the right track in recognizing false alarms.”
Attitude	FGR	“How skeptical we were about continuous monitoring… And now, three years later we cannot work without it.”
	R1	“Some patients will be discharged soon, for example today or tomorrow. Why should you still monitor all vital signs and check trends?”
	FGR	“Sometimes, a saturation drops during the night as it also does at home. We check on the patient because of this saturation drop and then the patient seems to be okay.”
Factors associated with patients	P2	“I like the idea of knowing my own vital signs, so that I know what I can expect. Firstly, I looked very often, but that became gradually less often.”
Internal aspects	R2	“I think, for example, during evening and nightshifts we are much of our time present in the nursing office. During that shift, we do not need a dedicated nurse. During day shifts, when everyone is at the patient’ rooms, I think a dedicated nurse is necessary. The question rises if you can deploy the dedicated nurse in patient care, and that he also receives all alarms, so that he can respond to the alarms.”
	FGR	“I would like to maintain continuous monitoring, but I would also like to retain total care for my patients, without shifting tasks. That is very important to me”.
	R3	“If the dedicated nurse signals a certain trend, and the nurse is not yet at the patient, then he [dedicated nurse] must inform me, so that I can visit the patient. If I [as nurse] do not get out, I can call the dedicated nurse to monitor trends.”
Factors associated with peers	R4	“We can say that connecting the device can be a task for nurse assistants. It will give a nice touch to their job profile. It seemed that some nurse assistants do really like that, and they see it as a challenge.”
External aspects	R3	“The difference between a high care ward and our general ward is getting smaller using this system. Subsequently, it is difficult to set boundaries, and to frame, between what you should do and not do.”

P = Patient; FGR: Focus group Respondent R: Respondent.

## Data Availability

The data presented in this study are available on request from the corresponding author.

## Supplementary Materials

The following supporting information can be downloaded at: https://www.mdpi.com/article/10.3390/jcm13020439/s1, Supplementary S1: Graphical presentation of the Visi Mobile; Supplementary S2: interview guide; Supplementary S3: Model of Gagnon.
