# Supplementary material for: Three Years of Continuous Vital Signs Monitoring on the General Surgical Ward: Is It Sustainable? A Qualitative Study
Source: J Clin Med. 2024 Jan 13;13(2):439. doi: 10.3390/jcm13020439 (PMC10816891; doi:10.3390/jcm13020439)
Supplement: Supplementary file 1 [file jcm-13-00439-s001.zip › Sup S1 - Graphical presentation of the Visi Mobile.pdf]

Supplementary S1. Graphical presentation of the Visi Mobile

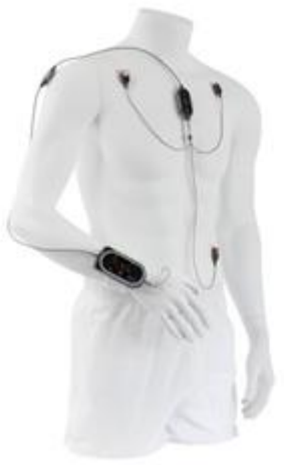

Figure S1. the VisiMobile (Sotera Wireless) having sensors on the chest and thumb, and a battery on the wrist.

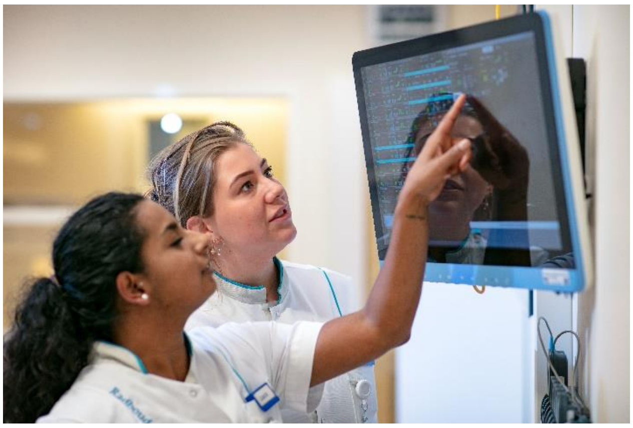

Figure S2. nurses assess the vital signs at a monitor in the nursing station (nurses gave permission for publication)
