# Supplementary material for: Three Years of Continuous Vital Signs Monitoring on the General Surgical Ward: Is It Sustainable? A Qualitative Study
Source: J Clin Med. 2024 Jan 13;13(2):439. doi: 10.3390/jcm13020439 (PMC10816891; doi:10.3390/jcm13020439)
Supplement: Supplementary file 1 [file jcm-13-00439-s001.zip › Sup S2 - Interview guides.pdf]

Supplementary S2. Topic guides for individual and focus group interviews.

Content:

A. Topic guide for key-user nurses
B. Topic guide for patients
C. Topic guide for focus group

<u>1A - Interview guide for key-user nurses</u>

Demographic characteristics

- Gender
- Age
- Educational level
- Experience

Introduction

-Introduction of interviewer and respondent

-Aim of the interview: We are interested in your perspective on how the use of continuous monitoring can be improved based on your experiences.

-The interview consists of open-ended questions and have four main topics: XX. During and at the end of the interview you can make additional remarks or ask questions.

-This interview will be recorded, if you consent. Please feel free to let us know if you have any doubts or questions before we start. The recording will be transcribed on paper, and your information will be kept anonymous. The interview will take about 15 to 30 minutes. Do you provide your consent for this interview? Do you have any questions?

<u>*Note for interviewer*</u>*: the questions below are helpful questions which can be asked when respondent does not have a satisfactory answer for the interviewer, meaning that not every suggestion of questions within a topic must be asked. At least make sure that every topic is answered by the respondents.*

Topics

Experiences with continuous monitoring

- How do experience the use of continuous monitoring?
- What are the advantages in your perspectives while use a system for continuous monitoring?
- When the sky is the limit, what new innovation would you want or change regarding continuous monitoring and the Visi Mobile?

Experiences regarding barriers

- What barriers do you experience at the moment regarding continuous monitoring?
- To what extent do you still recognize the barriers in box 1? *The barriers and artefacts in the use of the Visi Mobile are found in interviews during the study of Weenk et al, and in surveys during the study of Becking-Verhaar et al.. These studies were done in our hospital and this particular unit.*
- What are the most important barriers for you at this moment based on the problems that you mentioned and that are described in box 1?

Box 1: Barriers and artefacts in the use of the Visi Mobile for continuous monitoring

Findings from Weenk et al.	Findings from Becking-Verhaar et al.
• Information overload<br>• False and irrelevant alarms<br>• Uncomfortable for patients to see their vitals<br>• Battery replacement<br>• Release of plasters<br>• Patients are sometimes not sufficiently connected<br>• Nurses are not well educated to interpret all the data<br>• Insufficient response to alarms	• Visi Mobile is sometimes dysfunctional; parts are broken<br>• Insufficient WiFi connection<br>• Short battery life, up to 12 hours<br>• Administration of the device can be time consuming when problems occur<br>• Battery is too big and heavy<br>• Not well-trained nurses regarding Visi Mobile use<br>o Not all nurses have had the same basic education<br>o Errors are not solved because a lack of know-how

Possible improvements

- What could be possible improvements or innovations to solve these barriers in your perspective?
- How do you perceive that such an innovation could work? What does it mean for you and your work?
- What new innovation would you want or change regarding continuous monitoring and the Visi Mobile?
- What is your perspective towards an innovation within the way nurses work with continuous monitoring?
- How do you think that a dedicated nurse for continuous monitoring can solve the barriers that you have mentioned? *A dedicated nurse focusses on specific area or task, in this case on continuous monitoring. This particular nurse will deal with all kind of tasks and activities that are related to the device or continuous monitoring.*

Preconditions

- What preconditions do you foresee for the improvements or innovations? Think about education, professional space,
- What activities should be assigned to a dedicated nurse?
- How do you think this role can cooperate with other nurses, yourself, and medical doctors?
- What is required to apply the improvement that you mentioned or role in daily activities?

Closings

- If you would mention the most important innovation regarding continuous monitoring of vital signs, what would it be?
- If you would maintain something about the current way of using the system for continuous monitoring of vital signs, what would it be?
- Do you have any remarks, suggestions or questions?

Thank you very much!

<u>1B – Interview guide for patients</u>

Demographic characteristics

- Gender
- Age

Introduction

-Introduction of interviewer and respondent

-Aim of the interview: the aim of the interview is to investigate the perspectives of patients towards the Visi Mobile as a system for continuous monitoring. We ask for positive and negative aspects of it. We also will ask about your perspective towards innovations or improvements such as a dedicate nurse for continuous monitoring.

-This interview will be recorded, if you consent. Please feel free to let us know if you have any doubts or questions before we start. The recording will be transcribed on paper, and your information will be kept anonymous. The interview will take about 15 to 30 minutes. Do you provide your consent for this interview? Do you have any questions?

<u>Note for interviewer</u>: the questions below are helpful questions which can be asked when respondent does not have a satisfactory answer for the interviewer, meaning that not every suggestion of questions within a topic must be asked. At least make sure that every topic is answered by the respondents.

Topics

Experiences with continuous monitoring

- How do experience the use of continuous monitoring?
- What are the advantages or positive aspects in the use this system for continuous monitoring in your perspectives?
- What barriers do you experience at the moment regarding continuous monitoring?

Improvements

- What new innovation would you want or change regarding continuous monitoring and the Visi Mobile?
- How can such an innovation or improvement be integrated in daily routines?

- What is your perspective towards an innovation within the way nurses work with continuous monitoring?
- How do you think that a dedicated nurse for continuous monitoring can solve the barriers that you mention? *A dedicated nurse focusses on specific area or task, in this case on continuous monitoring. This particular nurse will deal with all kind of tasks and activities that are related to the device or continuous monitoring.*
- What activities should be assigned to a dedicated nurse? How do you think this ole can cooperate with other nurses, yourself, and medical doctors?
- How do you perceive that such a new role can benefit your health? What is in it for you?

Closing

- Do you have any remarks, suggestions or questions?

Thank you very much!

<u>1C – Interview guide for focus group with nurses</u>

Demographic characteristics

- Gender
- Age
- Educational level
- Experience

Introduction

-Introduction of interviewer and respondent

-Aim of the interview: the aim of this interview is to explore your perspectives towards the use of continuous monitoring using the Visi Mobile. This system is now used for some years in this ward, and we look to further improve its use and innovate our work of continuous monitoring.

Please be aware that every answer you have is relevant and important, it is your perception that we would like to hear.

-The interview consists of open-ended questions regarding your current experiences, identification of barriers or problems, and explore solutions or innovations to make improvements in the use of the continuous monitoring system. During and at the end of the interview you can make additional remarks or ask questions.

-This interview will be recorded, if you consent. Please feel free to let us know if you have any doubts or questions before we start. The recording will be transcribed on paper, and your information will be kept anonymous. The interview will take about 60 to 90 minutes. For the sake of transcription of the audio, please wait until someone is done taking before you bring in your idea's or response. Do you provide your consent for this interview? Do you have any questions?

<u>Note for interviewer</u>: the questions below are helpful questions which can be asked when respondent does not have a satisfactory answer for the interviewer, meaning that not every suggestion of questions within a topic must be asked. At least make sure that every topic is answered by the respondents.

Topics

Experiences with continuous monitoring

- How do you experience the use of continuous monitoring?
- What are the advantages in your perspectives while use a system for continuous monitoring?
- When the sky is the limit, what new innovation would you want or change regarding continuous monitoring and the Visi Mobile?

Experiences regarding barriers

- What barriers do you experience at the moment regarding continuous monitoring?
- To what extent do you still recognize the barriers in box 1? *The barriers and artefacts in the use of the Visi Mobile are found in interviews during the study of Weenk et al, and in surveys during the study of Becking-Verhaar et al.. These studies were done in our hospital and this particular unit.*
- What are the most important barriers for you at this moment based on the problems that you mentioned and that are described in box 1?

Box 1: Barriers and artefacts in the use of the Visi Mobile for continuous monitoring

Findings from Weenk et al.	Findings from Becking-Verhaar et al.
• Information overload<br>• False and irrelevant alarms<br>• Uncomfortable for patients to see their vitals<br>• Battery replacement<br>• Release of plasters<br>• Patients are sometimes not sufficiently connected<br>• Nurses are not well educated to interpret all the data<br>• Insufficient response to alarms	• Visi Mobile is sometimes dysfunctional; parts are broken<br>• Insufficient WiFi connection<br>• Short battery life, up to 12 hours<br>• Administration of the device can be time consuming when problems occur<br>• Battery is too big and heavy<br>• Not well-trained nurses regarding Visi Mobile use<br>o Not all nurses have had the same basic education<br>o Errors are not solved because a lack of know-how

Possible improvements

- What could be possible improvements or innovations to solve these barriers in your perspective?
- How do you perceive that such an innovation could work? What does it mean for you and your work?

- What new innovation would you want or change regarding continuous monitoring and the Visi Mobile?
- What is your perspective towards an innovation within the way nurses work with continuous monitoring?
- How do you think that a dedicated nurse for continuous monitoring can solve the barriers that you have mentioned? *A dedicated nurse focusses on specific area or task, in this case on continuous monitoring. This particular nurse will deal with all kind of tasks and activities that are related to the device or continuous monitoring.*

Preconditions

- What preconditions do you foresee for the improvements or innovations? Think about education, professional space,
- What activities should be assigned to a dedicated nurse?
- How do you think this role can cooperate with other nurses, yourself, and medical doctors?
- What is required to apply the improvement that you mentioned or role in daily activities?

Closings

- If you would mention the most important innovation regarding continuous monitoring of vital signs, what would it be?
- If you would maintain something about the current way of using the system for continuous monitoring of vital signs, what would it be?
- Do you have any remarks, suggestions or questions?

Thank you very much!
