# Supplementary material for: Three Years of Continuous Vital Signs Monitoring on the General Surgical Ward: Is It Sustainable? A Qualitative Study
Source: J Clin Med. 2024 Jan 13;13(2):439. doi: 10.3390/jcm13020439 (PMC10816891; doi:10.3390/jcm13020439)
Supplement: Supplementary file 1 [file jcm-13-00439-s001.zip › Sup S3 - Gagnon's list of factors.pdf]

Supplementary S3. Model of Gagnon.

Table 1. List of factors related to the success or failure of ICT adoption [1].

Indicator	Subindicator
1. Factors related to ICT	
Design and technical concerns	
Characteristics of the innovation	
	Relative advantage (usefulness)
	Compatibility (with work process, values)
	Ease of use / complexity
	Triability
	Observability
System reliability	
Interoperability	
Legal issues	
	Confidentiality - privacy concerns
	Other legal issues (including security)
Evidence regarding benefits of IT	
Validity of the resources	
	Scientific quality of the information resources
	Content available (completeness
	Appropriate for the users (relevance)
Cost issues	
Environmental issues	
2. Individual factors of healthcare professionals	
Knowledge	
	Awareness of the existence and/or objectives of the ICT
	Familiarity with ICT
	Familiarity with technologies in general
Attitude	
	Agreement with the particular ICT
	Applicability to the clinical situation
	Confidence in ICT developer
	Challenge to autonomy
	Impact on clinical uncertainty
	Time consuming/ time saving
	Outcome expectancy (use of the ICT leads to desired outcome)
	Motivation to use the ICT (readiness) /resistance to use the ICT
	Self-efficacy (believes in one’s competence to use the ICT)
	Impact on professional security

Agreement with ICTs in general (welcoming/resistant

Socio-demographic characteristics

Age

Gender

Experience

Ethnicity

Other

3. Human environment

Factors associated with patients

Patients' attitudes and preferences regarding ICT

Patient/health professional interaction

Applicability to patients' characteristics

Other factors associated with patients

Factors associated with peers

Attitude of colleagues about ICT

Support and promotion of ICT by colleagues

Others factors associated with peers (relations between colleagues)

4. Organisational aspects

Internal environment

Characteristics of the structure of work

Setting of care (hospital, outpatient, primary care)

Practice size

Status (university/other, private/public)

Physician salary status and reimbursement

Work (nature of work)

Time constraints and workload

Work flexibility

Relation between different health professionals (including role boundaries, change in tasks)

Professional culture

Skill -Staff

Leadership

Staff issues (stability, shortage)

Resources availability

External environment

Financing of ICT / financial support

Interorganisational relations

Health care policies

1. Gagnon, M.P., Desmartis, M., Labrecque, M., Car, J., Pagliari, C., Pluye, P., et al. Systematic review of factors influencing the adoption of information and communication technologies by healthcare professionals. *J Med Syst.* **2012** *36*, 241–277.
